# Application of a Machine Learning Algorithm in Generating an Evapotranspiration Data Product From Coupled Thermal Infrared and Microwave Satellite Observations

**DOI:** 10.3389/fdata.2022.768676

**Published:** 2022-05-20

**Authors:** Li Fang, Xiwu Zhan, Satya Kalluri, Peng Yu, Chris Hain, Martha Anderson, Istvan Laszlo

**Affiliations:** ^1^Earth System Science Interdisciplinary Center, Cooperate Institute of Satellite Earth System Studies (CISESS), University of Maryland, College Park, MD, United States; ^2^Center for Satellite Applications and Research (STAR), National Environmental Satellite, Data, and Information Service (NESDIS), National Oceanic and Atmospheric Administration (NOAA), College Park, MD, United States; ^3^Marshall Space Flight Center, National Aeronautics and Space Administration (NASA), Huntsville, AL, United States; ^4^Hydrology and Remote Sensing Laboratory, U.S. Department of Agriculture (USDA), Beltsville, MD, United States

**Keywords:** evapotranspiration (ET), land surface temperature (LST), GOES-R, machine learning (ML), regression tree (RT)

## Abstract

Land surface evapotranspiration (ET) is one of the main energy sources for atmospheric dynamics and a critical component of the local, regional, and global water cycles. Consequently, accurate measurement or estimation of ET is one of the most active topics in hydro-climatology research. With massive and spatially distributed observational data sets of land surface properties and environmental conditions being collected from the ground, airborne or space-borne platforms daily over the past few decades, many research teams have started to use big data science to advance the ET estimation methods. The Geostationary satellite Evapotranspiration and Drought (GET-D) product system was developed at the National Oceanic and Atmospheric Administration (NOAA) in 2016 to generate daily ET and drought maps operationally. The primary inputs of the current GET-D system are the thermal infrared (TIR) observations from NOAA GOES satellite series. Because of the cloud contamination to the TIR observations, the spatial coverage of the daily GET-D ET product has been severely impacted. Based on the most recent advances, we have tested a machine learning algorithm to estimate all-weather land surface temperature (LST) from TIR and microwave (MW) combined satellite observations. With the regression tree machine learning approach, we can combine the high accuracy and high spatial resolution of GOES TIR data with the better spatial coverage of passive microwave observations and LST simulations from a land surface model (LSM). The regression tree model combines the three LST data sources for both clear and cloudy days, which enables the GET-D system to derive an all-weather ET product. This paper reports how the all-weather LST and ET are generated in the upgraded GET-D system and provides an evaluation of these LST and ET estimates with ground measurements. The results demonstrate that the regression tree machine learning method is feasible and effective for generating daily ET under all weather conditions with satisfactory accuracy from the big volume of satellite observations.

## Introduction

Evapotranspiration (ET) is one of the main components of the global and regional hydrological cycle (Sato et al., 1989; Rabin et al., 1990; Bastiaanssen et al., 1998a,b). The latent heat from evapotranspiration is also one of the most important components of the energy cycle because it is the largest energy source for the atmosphere and thus is significant for weather and climate formation (Wetherald and Manabe, 1988; Sato et al., 1989; Allen et al., 1998, 2005). Satellite remote sensing of ET has been applied to monitor regional and global droughts in recent decades and is probably the most practical and efficient approach to providing the observational ET data for numerical weather, climate, and hydrological prediction models.

Based on the Atmosphere-Land Exchange Inversion (ALEXI) model, NOAA-NESDIS has developed an operational Geostationary Operational Environmental Satellites (GOES) ET and Drought (GET-D) product system using thermal infrared (TIR) observations of the Baseline Imagers on GOES-13 and GOES-15 satellites (Zhan et al., 2016, 2019; Fang et al., 2019). This data product system is designed for model validation, data assimilation in numerical weather and water prediction models, and drought monitoring applications.

With the primary operational GOES satellites transitioning to GOES-16 and GOES-17, the Advanced Baseline Imagers (ABI) on the new generation of NOAA GOES satellites allow the GET-D system to be significantly enhanced with higher spatial resolution and better accuracy. The GET-D system has therefore been upgraded to generate ET data products using ABI observations at 2 km spatial resolution covering the continental United States (CONUS). Several scientific advances to the ALEXI model, the core of the GET-D system, have been made in the past years and have been integrated into the new system (Anderson et al., 1997, 2007, 2011).

Given that the current GET-D product is dependent on the availability of remotely sensed TIR observations, the ALEXI model can only be executed under clear-sky conditions. As the demand for an all-weather ET and drought monitoring product continues to grow, potential techniques have been explored to use other data sources complementing the GOES ABI observations.

Rapid progress in the fusion of artificial intelligence (AI) in numerous fields has been achieved in recent years. AI has shown its growing potential in the exploitation of massive satellite data and model estimates for applications in numerical weather prediction (NWP), data assimilation and other Earth and environmental sciences (Williams et al., 2016; Hall, 2019; Boukabara et al., 2021). Machine learning (ML), as an important subset of AI, has made significant advances in diverse applications in the Earth system and remote sensing. ML has been used in numerous ways to substitute traditional physical models to derive surface parameters from remotely sensed observations (Reichstein et al., 2019; Wang et al., 2019; Wimmers et al., 2019). The ML technique, especially the regression tree (RT) approach, is innovative and evolutionary, making full use of massive and dynamic data to determine the relationships and hidden patterns under different conditions. The ML-generated satellite products have shown similar behavior as those derived from traditional physical models (Boukabara et al., 2021).

Given the potential benefits from AI for Earth observations, the regression tree machine learning technique has been developed and evaluated in this study to merge GOES TIR observations with microwave (MW) and land surface model (LSM) simulations to derive temperature under both cloud free and cloud cover conditions. The objectives of this study include (1) the development of the regression tree model to generate merged LST maps under all weather conditions; (2) ingesting all-weather LST into the GET-D system to derive an all-weather ET product; (3) the validation of the newly derived LST and ET data sets based on multi-sources of *in-situ* LST and ET measurements.

## GET-D System and The ALEXI Model

### GET-D System

The GET-D product system was developed by NOAA NESDIS scientists to operationally generate ET and multi-weekly drought maps at 8 km spatial resolution over the North America domain from September 2016. As GOES satellites are transitioning to the GOES-R series, the GET-D system had to be upgraded by integrating GOES-16 and GOES-17 ABI thermal observations to enhance spatial resolution to 2 km (Fang et al., 2019). The upgraded system has very high consistency with the previous one, as the spatial correlation for the whole CONUS domain reaches as high as 0.95 over the testing period from July to October 2017 when both GOES-16 and GOES-13 are available. The enhanced ET product not only maintains the consistency with the current one, but also has the capability of capturing much better spatial detail. Details of system upgrading with GOES-16/17 ABIs can be found in Fang et al. (2019).

Since the GOES LST product can only be obtained under cloud-free conditions, the current GET-D system produces ET maps over clear-sky pixels only. In order to increase the spatial coverage of the ET product, the other major upgrade to the GET-D system has been to couple alternative data sources with GOES to allow the retrieval of surface fluxes under cloud cover. The details on the machine learning approach use to derive the all-weather LST and ET and the quality analysis of the new LST and ET product are reported in this paper.

### The ALEXI Model

The core of the GET-D system is the Atmosphere-Land Exchange Inversion (ALEXI) model (Anderson et al., 1997), which is an extension to the two-source energy balance (TSEB) model (Norman et al., 1995). Flux partitioning in the ALEXI model is guided by the mid-morning rise in surface temperature, which is then partitioned into soil and vegetation components based on the surface vegetation fraction (Anderson et al., 2007). The model is coupled with a simple one-dimensional atmospheric boundary layer (ABL) model for regional applications. The lower boundary condition is provided by satellite observed radiometric temperature at two morning hours, 1.5 h after sunrise and 1.5 h before noon. The ABL model then relates the rise in air temperature above the canopy during this interval and the growth of the ABL to the time-integrated influx of sensible heating from the surface, and ET is computed as a partial residual to the energy budget. The energy balance in ALEXI currently does not apply to snow covered surfaces, thus a snow mask based on the near real time snow cover data product of the Interactive Multi-sensor Snow and Ice Mapping System (IMS) is used (Fang et al., 2019). However, future modifications can incorporate snow energy balance (Kongoli et al., 2014).

The ALEXI surface flux estimates have been evaluated at the ALEXI pixel scale (several km) in comparison with ground-based data and demonstrated reasonable performance over a wide range of climatic and vegetation conditions (Hain et al., 2011; Anderson et al., 2013; Fang et al., 2016). A flux disaggregation technique (DisALEXI; Anderson et al., 2004) using higher resolution thermal imaging from Landsat to spatially downscale to the flux tower footprint scale (~100 m) enables direct comparison with ground observations, indicating root-mean square errors in daily ET retrievals on the order of 1 mm / day (Yang et al., 2017, 2020; Anderson et al., 2018, 2021; Knipper et al., 2020). The implementation of the ALEXI model in the current GET-D system utilizes thermal channel observations from geostationary satellites. Since the thermal channel is sensitive to clouds, the spatial cover of the current ET product is largely affected by cloud contamination. Therefore, possible solutions to fill in surface temperature over cloudy pixels at the two morning hours in the ALEXI model have been examined and a machine learning technique has been tested in this study to create all-weather LST maps.

## Data Sets

### GOES Observations

GOES-16/17 ABI observations in the thermal channel (Band#13; 10.35 μm) are the primary satellite inputs to the ALEXI model (Hodges and Michalsky, 2016). The upgraded GET-D system can directly integrate GOES-16/17 land surface (skin) temperature product in the Continental United States scanning mode. The GOES LST product over the CONUS domain provides hourly temperature estimates over cloud clear and probably clear pixels at a spatial resolution of 2 km.

When the GOES-16/17 LST product is not available, the GET-D system can trace back to the GOES-R Radiance L1b product. Atmospheric corrections are applied to the ABI brightness temperature observations in the longwave infrared spectral channels. The split-window technique is integrated in the GET-D system to apply atmospheric corrections similar to that used for the GOES-LST product, making these system inputs consistent and comparable. The derived land surface temperature is then adopted in the ALEXI model.

Both GOES-16/17 brightness temperature products and LST products are available at the Comprehensive Large Array-data Stewardship System (CLASS) of NOAA (NOAA CLASS; available online: https://www.avl.class.noaa.gov).

### Microwave Satellite Observations

The Advanced Microwave Scanning Radiometer 2 (AMSR2) onboard the GCOM-W1 provides highly accurate measurements of the intensity of microwave emission and scattering (Kawanishi et al., 2003). AMSR2 sensor visits two times per day (1:30 am and 1:30 pm local time) with more than 99% coverage of the earth every 2 days. AMSR2 observes the Earth with 6 different spectral bands and 2 polarizations (McCabe et al., 2005).

Our previous study showed the Ka-band brightness temperatures at V polarization (Vpol) have higher agreements with GOES LST than the H polarization (Zhan et al., 2017, 2018; Sun et al., 2019). Therefore, the AMSR2 L1B Brightness temperature product at Vpol is used in this study to fill in the surface temperature estimates over cloudy pixels. The L1B swath data are re-gridded to a global map in geographic (latitude/longitude) projection, followed by remapping to the CONUS domain. The pre-processing procedure is applied to both AMSR2 ascending and descending observations before transitioning to the regression tree models.

### LST Simulations From Land Surface Model

Land surface model (LSM) based LST simulations are one of the key inputs in our designed data mining models. Selection of an appropriate LSM LST product took into consideration of the accuracy and coverage of the data. The Climate Forecast System Reanalysis (CFSR) product is the third-generation reanalysis product operationally generated at the National Centers for Environmental Prediction (NCEP) (Saha et al., 2010, 2014; Dee et al., 2014). The CFSR implements an improved core model, an advanced assimilation schemes, and an enhanced atmosphere-land-ocean-sea ice coupling process. Studies have shown the CFSR has improved precipitation correlation with more realistic interannual variability and long-term trends (Wang et al., 2011). The CFSR temperature simulations are collected in our study to be integrated with regression tree modeling. The CFSR model provides 6-h reanalysis and 3-h forecast data at the resolution of 0.25° in GRIB format. The meteorological variables from CFSR model are extracted and preprocessed before taken by the core model. The pre-processing of the CFSR data set includes conversion of GRIB format to binary, the image re-gridding to the CONUS domain and the temporal interpolation to the two ALEXI model times (1.5 h after local sunrise and 1.5 h before local noon).

### *In-situ* Measurements for Evaluation

Multiple ground-based data sets are used to evaluate the performance of the data mining model. Data from the U.S. Atmospheric Radiation Measurement (ARM) and the Surface Radiation Budget Network (SURFRAD) were collected and used for LST validation. These two networks are among the most used networks for satellite based LST validation (Faysash and Smith, 1999; Sun and Pinker, 2003; Pinker et al., 2009) because of their operational availability, providing a large amount of data covering a long period of time.

The ARM Cloud and Radiation Testbed (ARM/CART) site, located at Lamont, Oklahoma (36.607°N, 97.489°W), provides direct skin temperature measurement at a temporal resolution of 30 min. The multi-filter radiometer at the ARM facility detects the diffuse/total upwelling irradiance, which is then converted to the skin temperature based on the NOAA/Atmospheric Turbulence and Diffusion Division algorithm (Peppler et al., 2001; Hodges and Michalsky, 2016). The uncertainty of the radiometer is about 5%.

The SURFRAD network records the surface long-wave radiation, which is converted to skin temperature according to the Stefan-Boltzmann law (Augustine et al., 2000, 2005). Since the *in-situ* fluxes measurements provide a high temporal resolution of 1 min, the time-matching can be very precise by choosing the closest *in-situ* observations at 1.5 h after sunrise and 1.5 h before noon corresponding to the ALEXI model.

As for ET validation data sources, AmeriFlux–the North, Central and South America part of the FLUXNET- is the most referred network measuring ecosystem CO_2_, water, and energy fluxes over CONUS. The standard flux measurement instrument of AmeriFlux is the eddy covariance (EC) tower. With help from our collaborators, data from about 24 AmeriFlux sites have been collected and processed for validation of GET-D ET outputs.

The EC system continuously measures fluxes every 30 min. In order to compare with daily estimates from the ALEXI model, the observed half-an-hour fluxes are integrated into daily estimates using 48 observations throughout the day. The integrated daily fluxes are corrected to enforce energy budget closure. There are multiple ways to force closure including the latent heat closure method, Bowen Ratio correction, etc. Twine et al. suggested “Bowen-ratio closure may be the most appropriate” method to correct ground observed flux components (Twine et al., 2000). The Bowen-ratio closure method assumes that the ratio of sensible to latent heat flux is correctly measured by the EC system so that individual latent flux values can be adjusted to balance the surface energy equation. After the latent heat flux component (energy units) is corrected, the ET (in mass units) is then calculated using the dynamic latent heat of evaporation. These ground observations of daily ET were used to evaluate the satellite-derived ET at 2 km. We note, however, that at some heterogeneous flux sites, the tower footprint may not be representative at the 2-km model pixel scale. This must be considered in the assessment of the statistical metrics of comparison (Section Evaluation of the MW-TIR-LSM Coupled ET Data Product).

## Development of Machine Learning Models to Couple Multiple LST Data Sources

### Multi-Sources Selection for Machine Learning Models

The Regression tree (RT) is a subset of the machine learning technique that enables computers to solve complex problems. It is one of the most broadly used tools available to identify the relationships among complex environmental data (De'ath and Fabricius, 2000). Our study uses the RT data mining method to automatically search patterns and relationships within the training samples among GOES TIR and candidate LST data sources.

**Figure 1:**
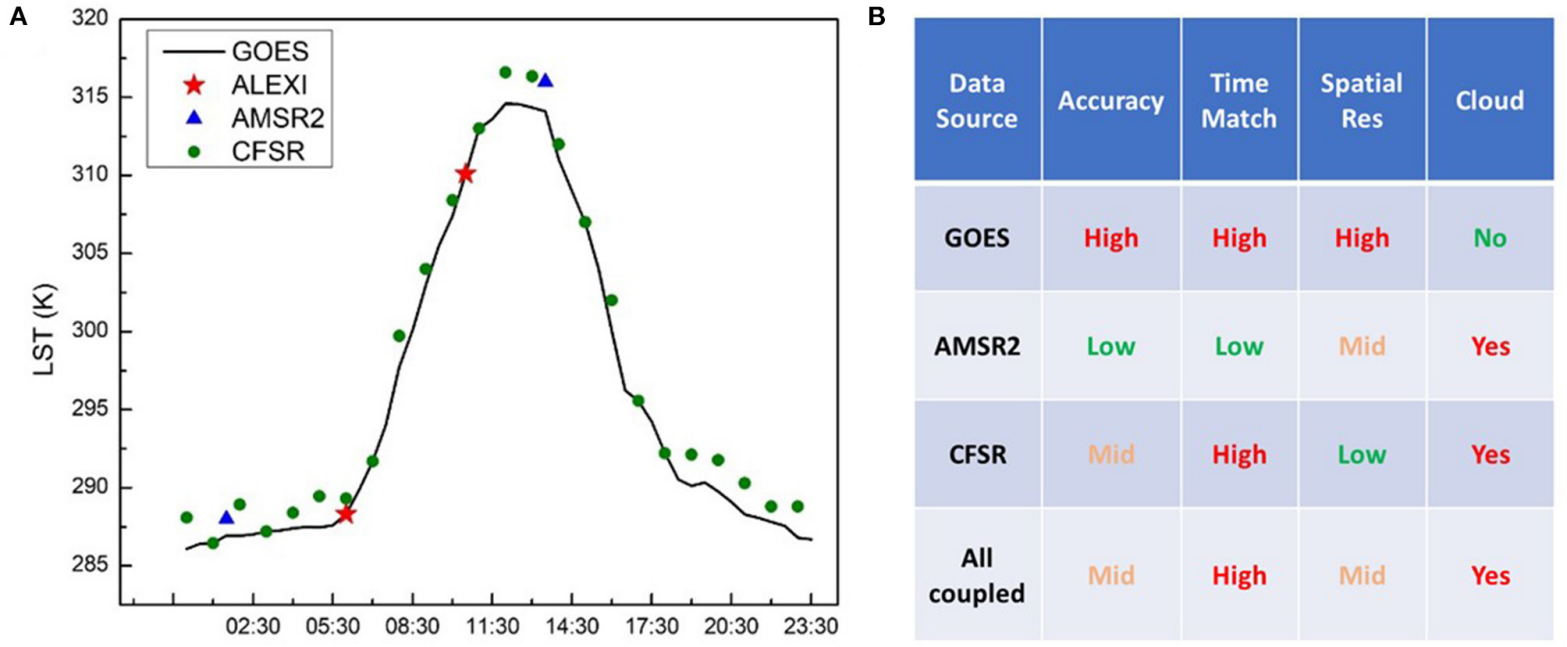
Comparison of different LST data sources for regression tree modeling in terms of their observational times **(A)** and their strengths and weakness **(B)**.

Before the design of ML RT model, the pros and cons of potential data sources were carefully weighed and analyzed. GOES ABI has a very high temporal resolution of every 5 min, being able to demonstrate diurnal variations of surface temperature as shown in Figure 1. Therefore, GOES ABI observations are well-suited to derive inputs to the ALEXI model at the two desired timestamps. Although GOES ABI has very high temporal and spatial resolutions with high accuracy, the biggest drawback is its sensitivity to the presence of clouds. Therefore, other data sources are needed to derive LST under cloudy conditions.

Studies have found a strong correlation between LST and the Ka-band brightness temperatures, with correlation coefficients reaching as high as 0.9 (Holmes et al., 2009). Microwave brightness temperature (TB) observations are largely unaffected by clouds and are generally easier to atmospherically corrected than thermal data. In this study, we use the Ka band (36.5GHz, v-pol) TB data of the Advanced Microwave Scanning Radiometer (AMSR2) on JAXA's GCOM-W1 satellite to derive LST for cloudy days. However, microwave observations have disadvantages as well. First, AMSR2 has relatively low temporal resolution with only two observations per day around 1:30 am and 1:30 pm local time. The two observational timestamps per day, which are around 5 h and 3 h apart from ALEXI morning rise hours, would inevitably introduce uncertainties in the regression model during the time matching. Second, due to lower signal strength in the microwave region, the accuracy and spatial resolution of AMSR2 observations are poorer than GOES observations, 9 km from AMSR2 vs. 2 km from GOES.

Microwave and thermal infrared channels complement each other, and our experimental results revealed that the regression model can predict LST within a reasonable range. The big concern, however, lies in the relatively weak representativeness of the training sets because GOES TIR cannot provide samples under cloudy conditions. This weakness motivated us to explore a third data source that could make up this shortcoming.

The land surface model-based LST has the advantages of full coverage and high temporal resolution although the spatial resolution is usually coarse. The characteristics of model temperature simulations make them well-suited to serve as a bridge for combining MW and TIR observations. The model-based LSTs can provide both cloud-free and cloudy samples, which is a good supplement to GOES TIR observations, and can better match ALEXI modeling hours, which can reduce the uncertainties in the time-matching between AMSR2 observational times and ALEXI morning hours.

Each data source has its own strengths and weaknesses which are outlined in Figure 1B, but by combining these three data sources together, we hypothesize we can generate LST maps at high resolution with high accuracy and most importantly covering all-weather conditions.

### Machine Learning Models to Couple MW, TIR and LSM LST Data

**Figure 2:**
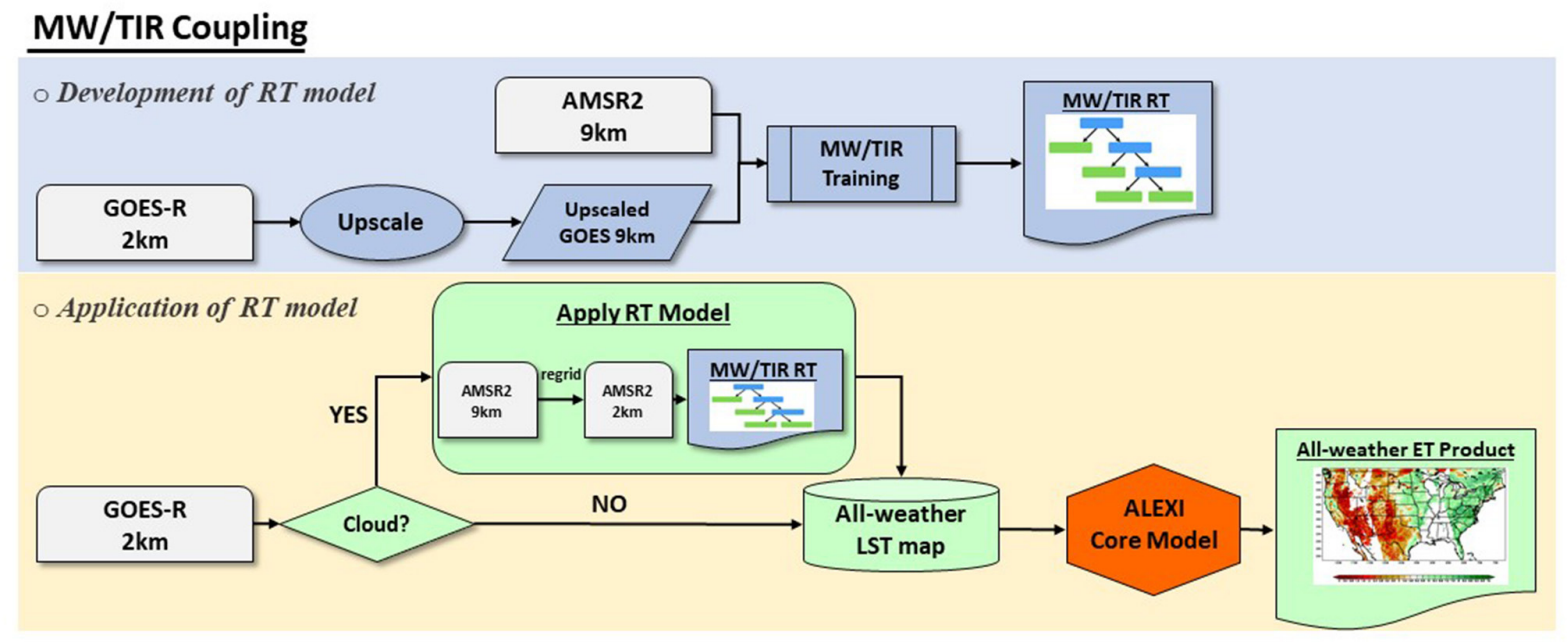
Data flow of the development of the regression tree model for merging microwave (MW) and thermal infrared (TIR) observations.

**Figure 3:**
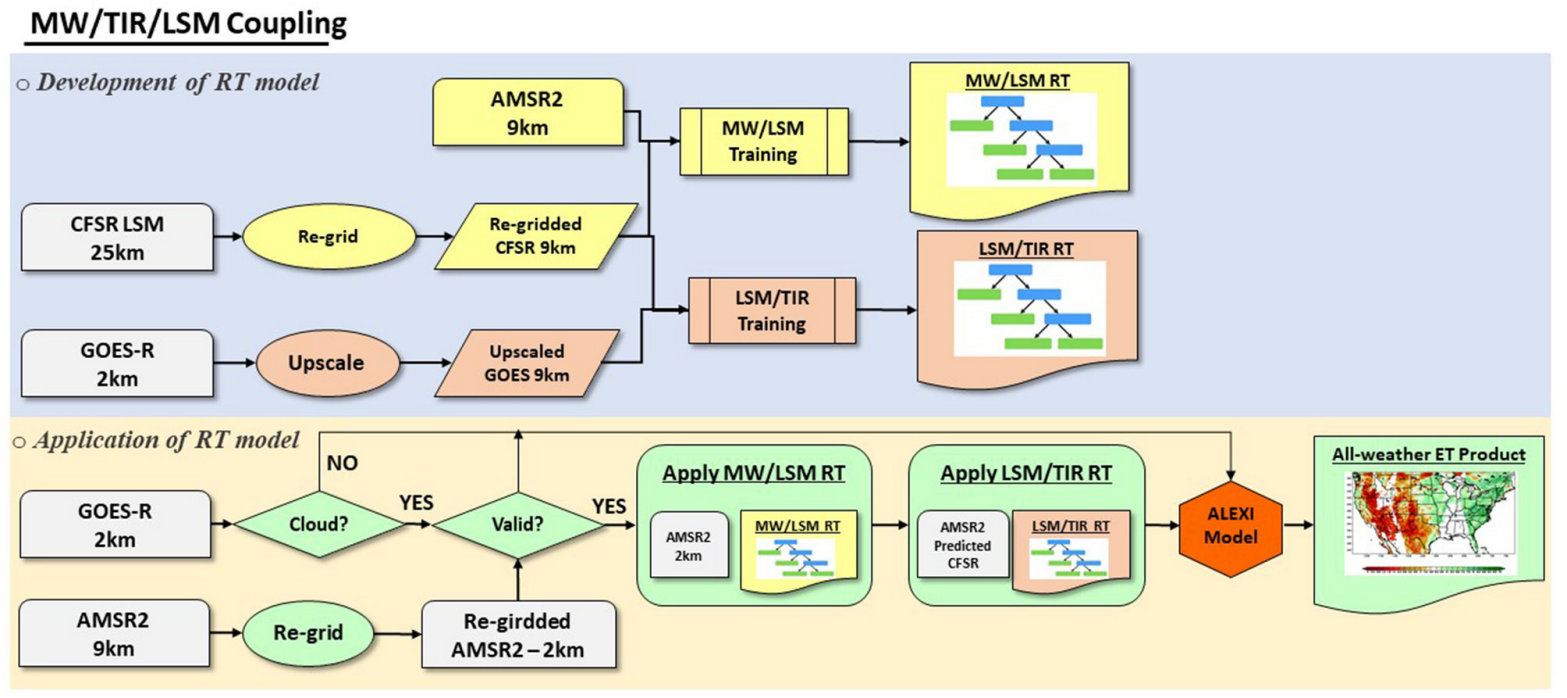
Data flow of the development of the regression tree model for merging microwave (MW), thermal infrared (TIR) and land surface temperature (LSM) estimates.

This study has examined two approaches to implementing the machine learning method for merging GOES-R LST with other data sources for all-weather coverage. The first is to directly build RT between GOES LST and AMSR2 TB (Figure 2), while the second is to use CFSR LST to connect AMSR2 and GOES-R observations (Figure 3). The coupling process includes two steps: the development of the regression tree (structure, coefficients, etc.), and the application of the RT model for prediction.

The M5 Model tree and rules are used in our experiments to build a regression tree from training samples. The original M5 algorithm was invented by Quinlan (1992) with improvements made by Wang (1997). The M5 Model first grows an unpruned decision tree by recursively splitting the instance space to maximally reduce the subset variation in the class variable. Post-pruning is applied to deal with the overfitting problem by generating an unpruned and complex tree first and simplifying it afterwards.

The processing procedure of the first approach for directly merging MW and TIR observations is presented in Figure 2. The regression tree is built for each pixel at the resolution of 9 km matching the scale of the AMSR2 brightness temperature product. The high resolution GOES-16/17 observations are first upscaled to 9 km using the simple average resampling method. The training samples are then collected between AMSR2 brightness temperatures and GOES-16/17 TIR observations. Quality control flags for each of the inputs data sets have been carefully checked in order to reduce the uncertainties of training samples. Once the regression tree model is built, it is applied to AMSR2 brightness temperature at Ka band to predict LST when GOES LSTs are not available. An all-weather LST map is then obtained by combining predicted AMSR2 LST and GOES-R LST retrievals, which is then integrated into the ALEXI core model to derive ET estimates.

Figure 3 demonstrates the data flow of the RT development coupling all three data sources. Two regression trees are built on the scale of AMSR2 product at 9km spatial resolution. The first training database is built between AMSR2 brightness temperatures and CFSR LST at the ALEXI model time 1 and time 2 of the day for both cloudy and cloud free scenarios. The first RT model is then created from the training data set to project AMSR2 observations to CFSR LSTs. A second RT is then developed between CFSR LST estimates and GOES-R LSTs for clear days. Assuming the clear day RT applies to cloudy days, it is then applied to estimate LST over cloudy cases. The representativeness of the training data sets is significantly enhanced with the integration of CFSR LST estimates by covering both clear-sky and cloudy samples and with better time-matching with the ALEXI model hours.

It is worth noting that even though the cloudy region that is filled by this method is at a 2 km grid resolution, the actual resolution is 9 km inherited from original AMSR2 TB observations. Future research would explore fusion or sharpening algorithms to downscale AMSR2 TB to a real 2 km spatial resolution before merging with GOES-R TIR observations. In that way, the all-weather LST map would have a consistent spatial resolution of 2 km.

The all-weather LST and ET predictions are evaluated by comparing them with *in-situ* measurements. The two coupling methods are inter-compared to analyze the benefit of the integration of model-based LST. The intercomparison results are presented in Section MW-TIR-LSM Coupled Method Compared with MW-TIR Method. Comprehensive evaluation of all-coupled LST and ET retrievals are given in Section Evaluation of the MW-TIR-LSM Coupled LST Retrievals and Section Evaluation of the MW-TIR-LSM Coupled ET Data Product, respectively.

## All-Weather LST/ET Retrievals and Evaluation Results

This section first evaluates the performance of the two machine learning methods. The benefit of introducing land surface model based LSTs into the regression tree model is analyzed by comparing the accuracy of LST/ET estimates from these two approaches. Following that, the quantitative evaluation of the final all-weather LST/ET data products is conducted. The study domain covers the CONUS domain over the period extending from July 2017 to July 2019, with an intensive evaluation period targeting the year of 2018. The all-weather LST retrievals at the two ALEXI model times are validated against *in-situ* measurements after time-matching and the daily all-weather ET product is compared with *in-situ* daily ET measurements. Time series comparisons over sample sites and overall error statistics are given in this section to evaluate the accuracy of the satellite retrievals.

### MW-TIR-LSM Coupled Method Compared With MW-TIR Method

The accuracy of LST retrievals derived from microwave and thermal observations (AMSR2 and GOES16/17) and three types of data sources (AMSR2, GOES16/17 and CFSR) are intercompared with *in-situ* LST measurements. Additionally, two separate GET-D experiments have been carried out to use LST data sets based on two-source coupling (MW/TIR) and three-source combined (MW/TIR/LSM) separately. The relative accuracy of corresponding ET retrievals is analyzed by comparing with ground daily ET measurements as well. The F-test has been applied to the MW/TIR coupling and MW/TIR/LSM combined retrievals with an alpha level of 0.05. The differences between these two data sets have passed the statistical significance test at 95% confidence level (statistical results are provided in the Supplementary Material).

**Figure 4:**
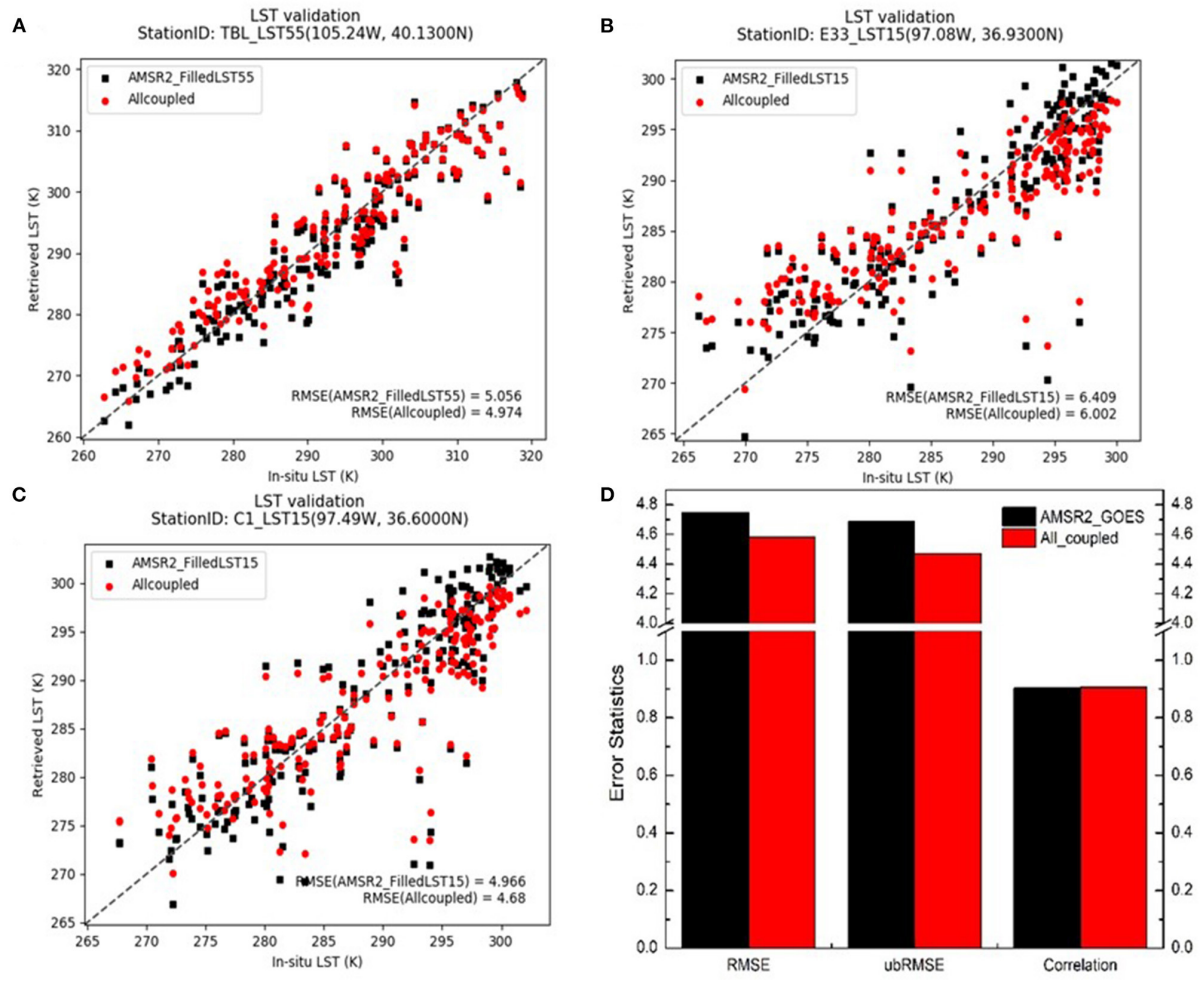
Validation of machine-learning-derived LSTs against *in-situ* LST measurements over the period from Jan. 26 to Dec. 31, 2018; **(A–C)** scatter plots of LSTs derived from MW/TIR (black) compared with those from MW/TIR/LSM (red) over three sample stations; **(D)** overall error statistics averaged from all validation sites.

As for the LST evaluation, scatterplots of machine-learning-derived LST against *in-situ* measurements over three ground stations are shown in Figures 4A–C, with the overall mean statistics averaged all measurements over the period from Jan. 26 to Dec. 31 in 2018 shown in Figure 4D. The level of agreement varies from site to site, depending in part on retrieval error and in part on sub-pixel heterogeneity. The agreement of derived LSTs is much higher at TBL station in Colorado than that at the other two sites (E33 and C1) in Oklahoma.

There are a handful of satellite LST retrievals underestimating the surface temperature by around 10K at the two sites in OK. They are caused by extremely low observed AMSR2 brightness temperatures of those samples. Adding CFSR LST in the regression tree model reduced the cold bias at those points by about 2K.

The ubRMSE of LST derived from combined AMSR2 and GOES16/17 is 4.69K, while the LST RMSE from MW/TIR/LSM coupling method is about 4.47K. The introduction of CFSR model based LST decreased the ubRMSE by 4.7% over the study period. The statistics also show reduced RMSE and enhanced correlation after incorporating CFSR LSTs into the regression tree model.

Two experimental GET-D runs were set up to use MW/TIR coupled LST and MW/TIR/LSM combined LST separately and the corresponding ET estimates were evaluated by comparing with *in-situ* measurements. The RMSE and correlation of the two sets of ET retrievals are listed in Table 1. The ET estimates based on all-coupled LST (MW/TIR/LSM) have the RMSE of 1.80 mm/day on average, 6.3% lower than those from MW/TIR coupled LST as inputs. The validation results show that the correlation between MW/TIR coupled ET estimates and *in-situ* is 0.67, while the r of three-source coupling method reaches 0.71. Both data sets present decent agreement with *in-situ* measurements with the all-coupled method having slightly higher correlation”.

**Table 1:** Error statistics (mean RMSE and correlation) of ET retrievals derived from MW/TIR and MW/TIR/LSM methods validated against AmeriFlux sites from Jan. 26 to Dec. 31, 2018.

**ET Products**	**RMSE (mm/day)**	**Correlation**
ET from coupled MW/TIR	1.93	0.67
ET from coupled MW/TIR/LSM	1.80	0.71
Improvement	6.74%	5.97%

Machine learning algorithms exploit maximum information from all data sources to build the relationship within the training samples. Satellite microwave and thermal channels provide complementary information, while land surface model LST simulations play a unique role in connecting them. The comparative analysis on both LST and ET evaluations indicates the added value of integrating model-based LST into the ML model is significant. LST and ET estimates from the all-coupled approach show better agreement with *in-situ* observations with relatively lower RMSE/ubRMSE and higher correlation, compared with those derived from MW and TIR channels. Therefore, the all-coupled method (MW/TIR/LSM) is chosen in our upgraded GET-D system to generate the final all-weather ET product. The following Sub-Sections (Evaluation of the MW-TIR-LSM Coupled LST Retrievals and Evaluation of the MW-TIR-LSM Coupled ET Data Product) will focus on the validation of GET-D outputs from the MW/TIR/LSM coupling method.

### Evaluation of the MW-TIR-LSM Coupled LST Retrievals

**Figure 5:**
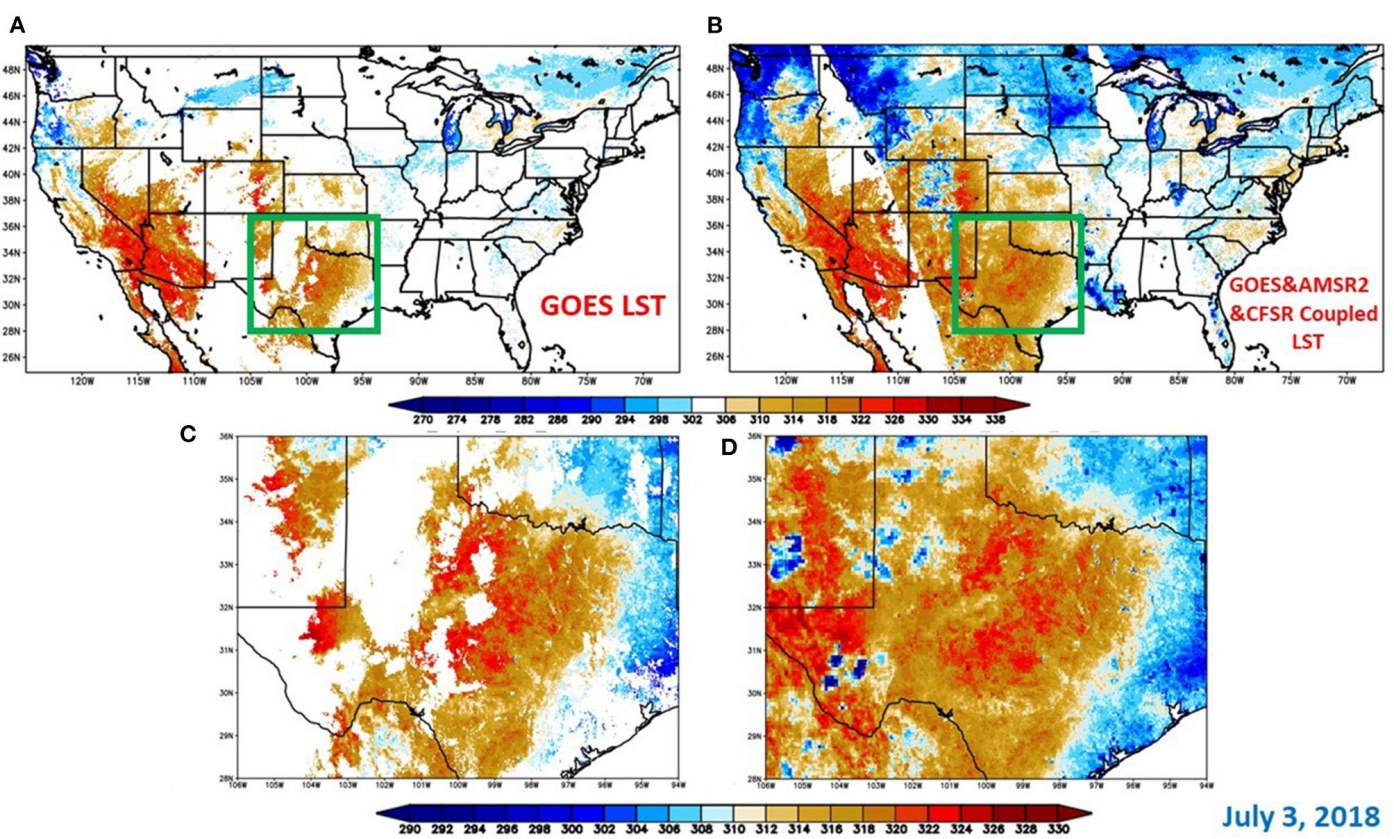
Visual comparison between GOES LST **(A,C)** and MW/TIR/LSM coupled LST **(B,D)** at ALEXI Time 2 (1.5 local hour before noon); **(A,B)** over the CONUS domain and **(C,D)** over the Lower Mississippi Valley region; July 3, 2018.

The MW/TIR/LSM coupled LSTs derived by the regression tree models were analyzed qualitatively and quantitatively in this section. A visual comparison of the clear-sky LST based on GOES ABI and all-weather LST from the all-coupled method is shown in Figure 5 over the CONUS domain (Figures 5A,B) and the Texas region (Figures 5C,D) on July 3, 2018. The improvement in data coverage is very notable. Results show the relative improvement in the data coverage increases by 260% averaged over the CONUS domain in 2018. This reduces the number of pixels that need to be filled by interpolation, providing better ability to capture rapid changes in surface moisture conditions. It is encouraging to see that the general pattern over the CONUS domain is reasonable with a smooth transition between cloud-free and cloud regions.

**Figure 6:**
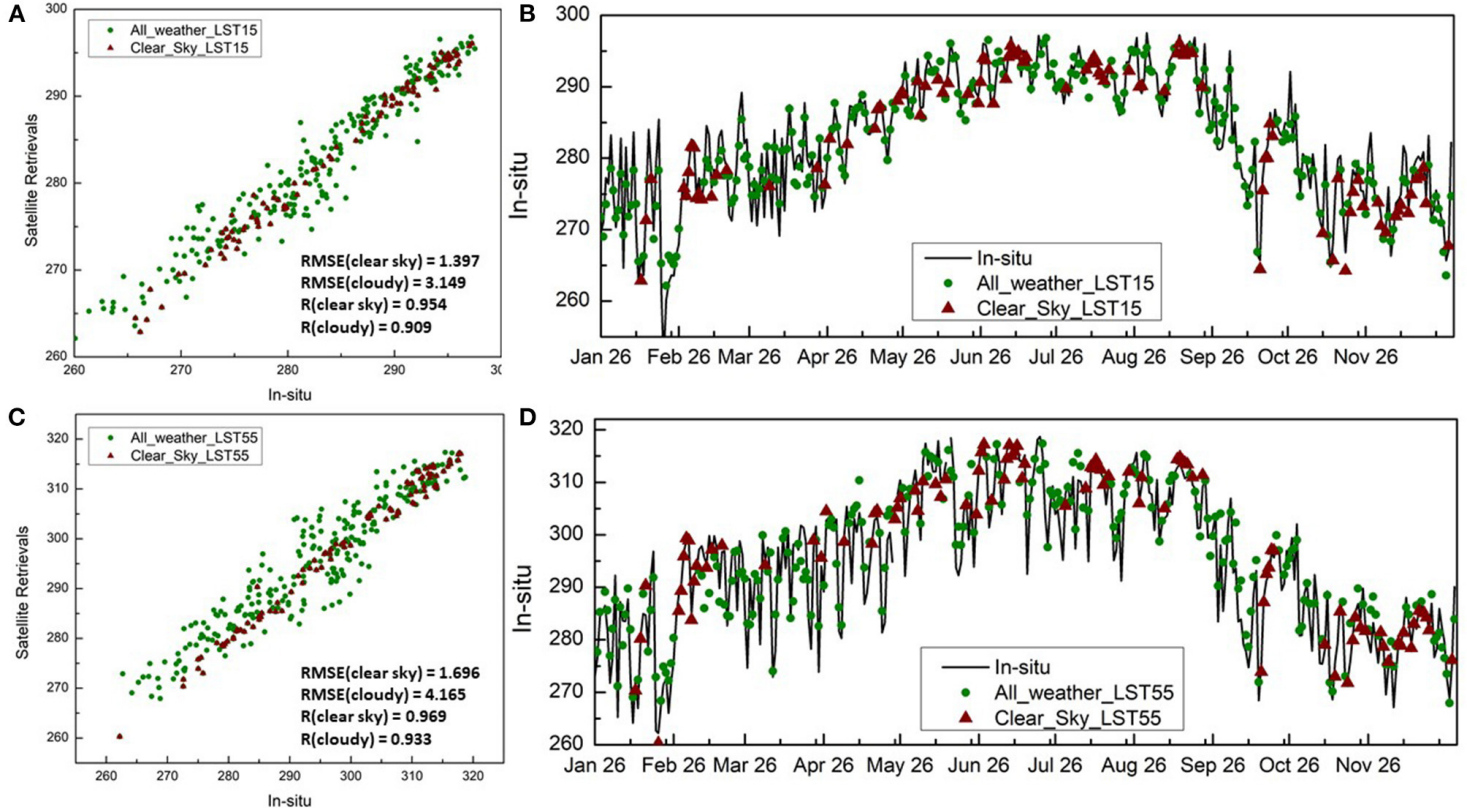
Scatter plot **(A,C)** and time series **(B,D)** comparison of clear-sky LST (based on GOES16/17 only) and all-weather LST (based on combined GOES/AMSR2/CFSR), along with *in-situ* LST measurements over the SURFRAD-TBL station; **(A,B)** comparison at Time 1 (1.5 hour after sun rise) and **(C,D)** comparison at Time 2 (1.5 hour before noon); Unit: K.

The accuracy of the all-weather LST retrievals was further examined using LST *in-situ* measurements from 6 sites over CONUS. The time series comparisons of clear-sky LST (based on GOES16/17) and all-weather LST (based on combined GOES/AMSR2/CFSR) over each of the validation site are provided in the Supplementary Material. One example over the SURFRAD-TBL site is presented in Figure 6, showing comparisons at ALEXI time 1 (Figures 6A,B) and time 2 (Figures 6C,D). The temporal data coverage increases by more than 200% for both ALEXI model times at the SURFRAD-TBL station over the validation period in 2018. The scatter plots in Figures 6A,C indicate that LST retrievals under cloudy days do not show significant bias with the *in-situ* measurements and the correlation coefficients at morning hour and noon hour reach 0.84 and 0.91, respectively. The time series comparison illustrates that the coupled LST retrievals can better catch the daily fluctuation after filling in the cloudy days. The all-weather LST predictions agree well with the *in-situ* LST measurements for both warming-up and cooling-down trends.

The error statistics of RMSE and correlation are shown in Tables 2, 3, respectively. The LST retrievals agree with the *in-situ* observations better on cloud-free days than cloudy days. Average RMSE and correlation coefficients (r) of the ABI only LST retrievals are respectively 1.2° and 0.95 for GET-D Time1 (1.5 h after sunrise) and 2.36° and 0.96 for Time 2 (1.5 hour before local noon). The corresponding RMSE and r values of the AMSR2 based LST retrievals are respectively 4.5° and 0.84 for Time 1 and 4.65° and 0.91 for Time 2, respectively. It is understandable that clear-sky retrievals have lower RMSE and higher correlation because the uncertainties under cloudy conditions are higher compared to clear sky situations. In general, the quality of all-weather LST retrievals is reasonable and acceptable as the mean correlation under all conditions could reach as high as 0.91.

**Table 2:** RMSE of MW-TIR coupled LST validated against *in-situ* observations (Jan. 26–Dec. 31 2018); statistics of clear sky pixels and cloudy pixels are separated.

**RMSE**			**LST15**		**LST55**	
**Site ID**	**LAT**	**LON**	**Clear sky**	**Cloudy**	**Clear sky**	**Cloudy**
C1	36.6	−97.49	1.517	4.121	2.298	4.964
E12	36.84	−96.43	1.044	4.034	2.622	4.120
E33	36.93	−97.08	1.250	5.176	2.305	4.138
E41	36.88	−97.09	1.072	5.471	2.852	5.022
FPK	48.31	−105.1	0.888	5.137	2.386	5.494
TBL	40.13	−105.24	1.397	3.149	1.696	4.165
Average RMSE			**1.195**	**4.514**	**2.360**	**4.651**

**Table 3:** Correlation of MW-TIR coupled LST validated against *in-situ* observations (Jan. 26–Dec. 31 2018); statistics of clear sky pixels and cloudy pixels are separated.

**Correlation**			**LST15**		**LST55**	
**Site ID**	**LAT**	**LON**	**Clear sky**	**Cloudy**	**Clear sky**	**Cloudy**
C1	36.6	−97.49	0.953	0.862	0.952	0.865
E12	36.84	−96.43	0.966	0.851	0.963	0.907
E33	36.93	−97.08	0.937	0.755	0.945	0.910
E41	36.88	−97.09	0.936	0.734	0.940	0.878
FPK	48.31	−105.1	0.973	0.907	0.979	0.947
TBL	40.13	−105.24	0.954	0.909	0.969	0.933
Average Correlation			**0.953**	**0.836**	**0.958**	**0.906**

### Evaluation of the MW-TIR-LSM Coupled ET Data Product

The all-coupled LST data were used in the ALEXI model to derive ET under all weather conditions. The all-weather ET outputs were compared with the clear-sky ET product visually and quantitatively. The two sets of ET retrievals are examined against AmeriFlux field measurements over CONUS.

**Figure 7:**
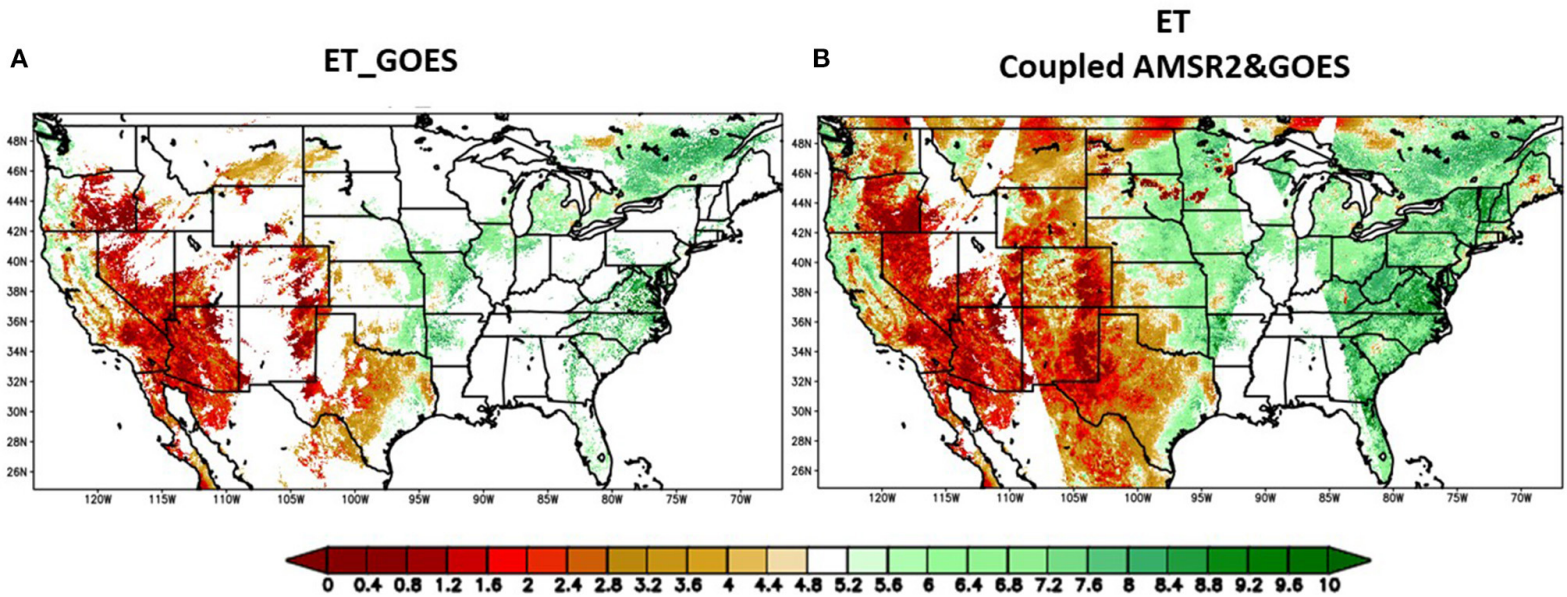
Visual comparison of clear-sky ET derived from GOES16/17 **(A)** and all-weather ET derived from GOES/AMSR2/CFSR combined LST **(B)** on May 30, 2018; Unit: mm/day.

One example of clear-sky ET from GOES-16/17 compared with the all-weather ET over CONUS domain on July 3, 2018, is shown in Figure 7. With the introduction of AMSR2 descending and ascending combined observations, the spatial coverage increases by around 260%. Most of the Great Plains and the Northern East Coast regions affected by the cloud have been filled in with reasonable patterns. Particularly, the states of Washington, North Dakota, South Dakota, Nebraska, and Minnesota are among the most cloud-contaminated areas and yet have been almost fully filled in by the all-weather ET map. The machine-learning predicted ET presents a general increasing trend from west to east across the CONUS domain, as expected.

**Figure 8:**
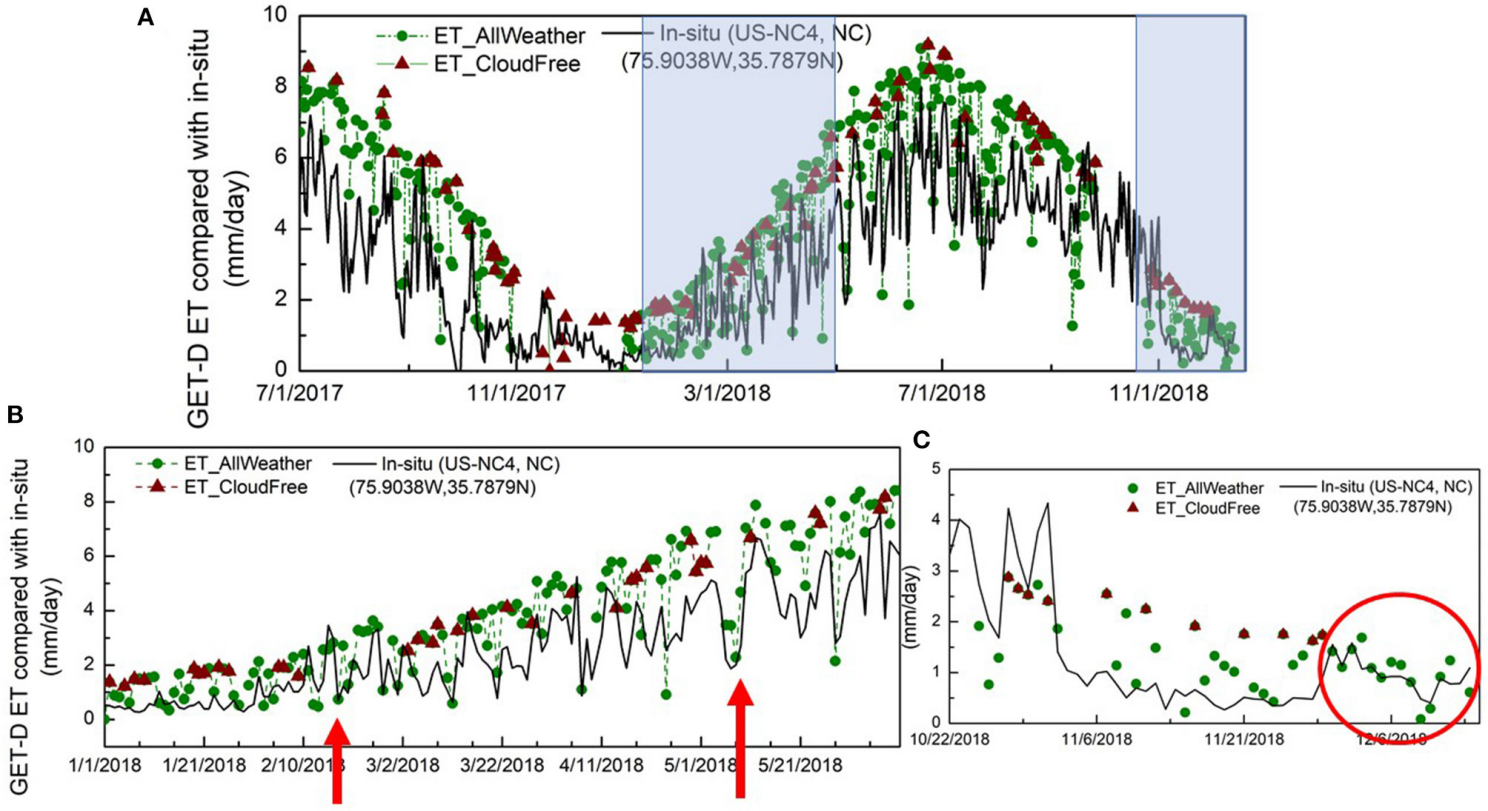
Time series comparison between clear-sky ET and all-weather ET, as well as *in-situ* ET observations at the AmeriFlux station in North Carolina; **(A)** over the period from July 1, 2017 to Dec. 31, 2018; **(B)** Jan.1 – June 10, 2018; **(C)** Oct. 22 - Dec. 14, 2018.

**Figure 9:**
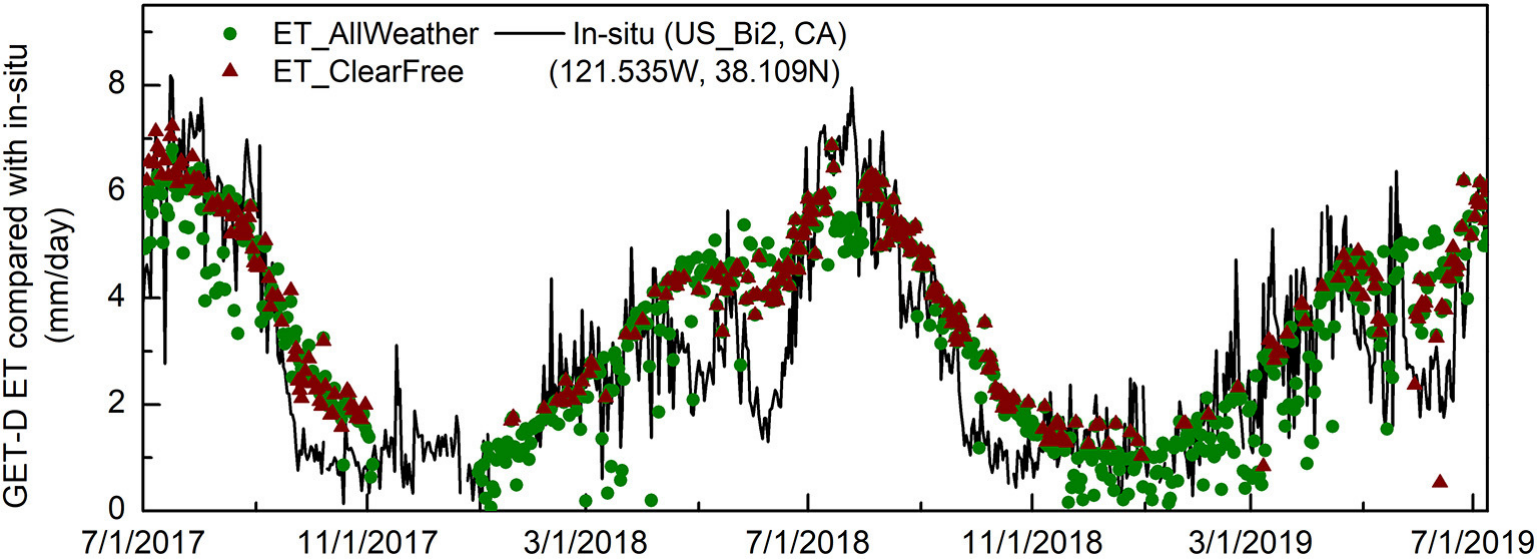
Same as Figure 8, but at the station (US-Bi2) in CA.

The all-weather ET retrievals were evaluated in comparison with *in-situ* ET measurements over the validation period from Jan. 1 to Dec. 31, 2018. Time series of comparison between the clear-sky and cloud-filled ET data sets are shown for two example sites in Figures 8, 9, with overall error statistics (RMSE and r) averaged from all validation sites over the CONUS domain provided in Table 4.

**Table 4:** Statistical comparison of GET-D ET estimates with *in-situ* measurements over a period from July 2017 to July 2019.

			**RMSE (mm/day)**		**Correlation**		**ubRMSE (mm/day)**		**MEAN ET (mm/day)**	
**Site ID**	**LAT**	**LON**	**Clear-sky**	**Cloudy**	**Clear-sky**	**Cloudy**	**Clear-sky**	**Cloudy**	**Clear-sky**	**Cloudy**
US-ARM	36.606	−97.489	1.090	1.276	0.836	0.703	0.994	1.234	2.712	2.635
US-Bi1	38.099	−121.499	1.611	1.384	0.599	0.739	1.518	1.332	4.389	3.478
US-Bi2	38.109	−121.535	1.178	1.205	0.828	0.792	1.095	1.179	4.108	3.413
US-Hn2	46.689	−119.464	1.745	1.097	0.432	0.565	1.002	0.820	2.761	1.878
US-IB1	41.859	−88.223	0.559	0.994	0.921	0.813	0.487	0.902	3.776	3.263
US-IB2	41.841	−88.241	0.544	0.834	0.972	0.856	0.497	0.826	3.496	3.177
US-KFS	39.056	−95.191	1.884	1.671	0.518	0.396	1.726	1.544	5.049	3.834
US-KLS	38.775	−97.568	1.690	1.630	0.091	0.486	1.065	1.261	3.496	3.120
US-MOz	38.744	−92.200	1.721	1.516	0.880	0.870	0.847	0.928	5.002	4.778
US-NC2	35.803	−76.669	1.944	2.104	0.919	0.808	0.931	1.299	6.067	6.009
US-NC3	35.799	−76.656	1.449	1.674	0.813	0.767	1.392	1.547	4.324	4.297
US-NC4	35.788	−75.904	1.803	2.142	0.844	0.786	1.031	1.340	6.027	5.708
US-NR1	40.033	−105.546	0.945	1.218	0.889	0.624	0.420	0.959	3.459	3.873
US-Rms	43.065	−116.749	1.343	1.273	0.682	0.611	1.173	1.272	3.009	2.730
US-Ro4	44.678	−93.072	1.788	1.580	0.732	0.723	1.117	1.171	3.977	3.386
US-Ro5	44.691	−93.058	0.915	1.143	0.824	0.737	0.903	1.143	3.790	3.112
US-Ro6	44.695	−93.058	1.403	1.088	0.544	0.752	1.401	1.077	3.775	3.100
US-Rws	43.168	−116.713	1.335	1.259	0.686	0.589	0.505	0.724	2.265	2.168
US-Sne	38.037	−121.755	0.832	1.322	0.897	0.895	0.684	0.955	3.566	3.750
US-SRG	31.789	−110.828	0.924	1.108	0.819	0.706	0.770	1.025	2.056	2.140
US-SRM	31.821	−110.866	0.711	0.933	0.796	0.701	0.695	0.920	1.844	1.952
US-Tw3	38.116	−121.647	1.378	1.274	0.541	0.664	1.163	1.115	3.770	3.411
US-Var	38.413	−120.951	2.288	2.265	0.353	0.568	2.185	2.035	3.145	2.793
US-WCr	45.806	−90.080	1.481	1.554	0.827	0.770	1.121	1.286	4.791	3.950
US-Whs	31.744	−110.052	1.244	1.242	0.705	0.605	0.527	0.887	2.183	2.223
US-Wkg	31.737	−109.942	1.600	1.475	0.757	0.593	0.636	1.112	2.582	2.629
**Average**			**1.362**	**1.395**	**0.719**	**0.697**	**0.996**	**1.150**	**3.670**	**3.339**

Figure 8 shows the time series comparison over a pine plantation site in North Carolina site from the AmeriFlux network. As shown in the figure, the wine triangles are the clear-sky ET from GOES 16/17 observations only, while green dots are the all-weather ET retrievals covering both clear and cloudy days. The all-weather ET retrievals better capture daily dynamics, providing good agreement with the *in-situ* records (Figure 8B). In Dec 2018, there was a period of about 2 weeks (Figure 8C) when the GOES thermal observations were missing due to persistent cloud cover, yet the all-coupled ET estimates provided reasonable predictions. Another example (US-Bi2; Figure 9) shows fluxes over an irrigated corn field in CA. While we do not expect perfect agreement due to the scale difference between the tower flux measurement and model pixel, the general envelope of the fluxes is well defined.

The relative accuracy of clear-sky ET is compared with that of all-weather retrievals in terms of the mean correlation coefficient and RMSE averaged from all the validation stations (Table 4). While this does not constitute an absolute model accuracy assessment, given the difference in scale between measurement and model pixel, it does suggest that the performance of the all-weather ET retrievals is similar to that of the traditional clear-sky method, with RMSE of approximately 1.4 mm/day and correlation of about 0.7. These performance metrics at 2-km resolution are lower than the ~ 1 mm/day accuracy reported when ALEXI is disaggregated to the tower footprint scale (RMSE ~ mm/day and R ~ 0.9; Anderson et al., 2018), better accounting for sub-pixel heterogeneity effects. Still, using the clear-sky ALEXI statistics as a benchmark, the relative performance of all-sky retrievals indicates that the machine learning approach can be effectively used to combine thermal, microwave, and model based LSTs to generate an ET product under all-weather conditions.

## Summary and Discussion

The GET-D system has been upgraded to provide high-quality ET estimates at the high spatial resolution of 2 km over the CONUS domain. Additionally, a RT machine learning approach has been developed to integrate microwave and LSM LST with GOES thermal observations to allow the retrieval of surface energy fluxes under cloud cover. This capability helps to fill in significant gaps in the cloud-free data product. The all-weather ET product increases data coverage by around 260% averaged over the CONUS domain in 2018 compared to the clear-sky ET product. The significantly improved data availability is imperative to promote applications of the new ET product.

Comparing to the commonly used microwave and thermal merging method, land surface model based LST simulations have been tested and integrated into the regression tree model. The benefits of the integration of CFSR LST estimates were quantitatively analyzed by comparing them with MW/TIR merging method. The evaluation results indicated that the use of CFSR LSTs helped to reduce the cold bias of the predictions on the order of 2K to 5K. The all-coupled LST estimates, which combine the strengths of satellite thermal and microwave channels and land surface models, agree well with *in-situ* LST measurements with the correlation coefficients at morning hour and noon hour of 0.836 and 0.906, respectively.

With the all-coupled LST inputs, the GET-D run was set up to generate the all-weather ET product over the testing period from Jan. 1 to Dec. 31, 2018. The quality of the all-weather ET product was further evaluated against more than 20 ground stations from the AmeriFlux network. It is promising to see that the all-weather ET retrievals not only match the annual trend with the *in-situ* records but also capture the daily fluctuations with cloudy days filled in. The overall statistics of the correlation and RMSE also illustrated the new ET product has the accuracy at the same level as the clear-sky product with the correlation coefficient of 0.7 averaged from all validation stations in 2018.

Although our preliminary results indicate that the regression tree machine learning can be used to merge different satellite data, challenges and improvements are worth further discussing. First, creating training sets is always a challenging topic for machine learning models because model predictions could be unreliable when extrapolating beyond the range of the training samples. In our experiments, highly uncertain observations have been removed from training samples according to the product quality control flags, but further investigations is needed on how to create representative and comprehensive training sets. Second, recent studies have shown that a better understanding of the underlying properties of ML models is imperative to leverage those techniques in the exploitation of satellite earth observations (McGovern et al., 2017; Samek et al., 2017). Future research should consider the development of an explainable and meaningful AI/ML system.

Future evaluation of the ET product will incorporate data from additional Ameriflux sites, but screening for sites that are more homogeneous at the 2-km pixel scale. The DisALEXI flux disaggregation method can also be employed to downscale to the flux tower footprint over more heterogeneous sites. Finally, the operational pathway of the upgraded GET-D at the Office of Satellite and Product Operations (OSPO) of NOAA or the Cloud will be investigated and identified in the near future.

## Data Availability Statement

The original contributions presented in the study are included in the article/Supplementary Material, further inquiries can be directed to the corresponding author/s.

## Author Contributions

XZ worked on system design, product validation and revised the manuscript. SK is responsible for project administration. PY provided and processed GOES-R LST products and prepared the in-situ LST validation data sets. CH and AM initiated the ALEXI model and worked on GET-D system design and implementation. IL provided the solar insolation input for GET-D. LF implemented the system upgrade, carried out validation and wrote the paper. All authors contributed to the article and approved the submitted version.

## Funding

Research of this study was supported by the NOAA NESDIS GOES-R Risk Reduction (GOES-R3) program for project #488.

## Author Disclaimer

The paper contents are solely the opinions of the authors and do not constitute a statement of policy, decision, or position on behalf of NOAA or the U. S. Government.

## Conflict of Interest

The authors declare that the research was conducted in the absence of any commercial or financial relationships that could be construed as a potential conflict of interest.

## Publisher's Note

All claims expressed in this article are solely those of the authors and do not necessarily represent those of their affiliated organizations, or those of the publisher, the editors and the reviewers. Any product that may be evaluated in this article, or claim that may be made by its manufacturer, is not guaranteed or endorsed by the publisher.
