# Supplementary material for: Application of a Machine Learning Algorithm in Generating an Evapotranspiration Data Product From Coupled Thermal Infrared and Microwave Satellite Observations
Source: Front Big Data. 2022 May 20;5:768676. doi: 10.3389/fdata.2022.768676 (PMC9163788; doi:10.3389/fdata.2022.768676)
Supplement: Supplementary file 1 [file Data_Sheet_1.docx]

Supplementary Material

**Supplementary** **Table 1.** F-Test Two-Sample for Variances (Alpha: 0.05)

|  | **E12** | | **E33** | | **E41** | | **FPK** | | **C1** | | **TBL** | |
| --- | --- | --- | --- | --- | --- | --- | --- | --- | --- | --- | --- | --- |
|  | **AMSR2**  **-filled** | **All-coupled** | **AMSR2**  **-filled** | **All-coupled** | **AMSR2**  **-filled** | **All-coupled** | **AMSR2**  **-filled** | **All-coupled** | **AMSR2**  **-filled** | **All-coupled** | **AMSR2**  **-filled** | **All-coupled** |
| Mean | 294.664 | 295.401 | 295.409 | 296.477 | 295.283 | 296.565 | 274.390 | 274.352 | 295.859 | 297.033 | 286.220 | 285.391 |
| Variance | 97.709 | 72.762 | 112.191 | 86.050 | 112.141 | 83.879 | 190.857 | 137.979 | 141.333 | 99.191 | 86.659 | 63.033 |
| Obs | 273 | 335 | 272 | 334 | 276 | 334 | 309 | 334 | 266 | 336 | 277 | 334 |
| df | 272 | 334 | 271 | 333 | 275 | 333 | 308 | 333 | 265 | 335 | 276 | 333 |
| F | 1.343 |  | 1.304 |  | 1.337 |  | 1.383 |  | 1.425 |  | 1.375 |  |
| **P(F<=f) one-tail** | **0.005** |  | **0.011** |  | **0.006** |  | **0.002** |  | **0.001** |  | **0.003** |  |
| F critical one-tail | 1.208 |  | 1.209 |  | 1.208 |  | 1.202 |  | 1.210 |  | 1.208 |  |


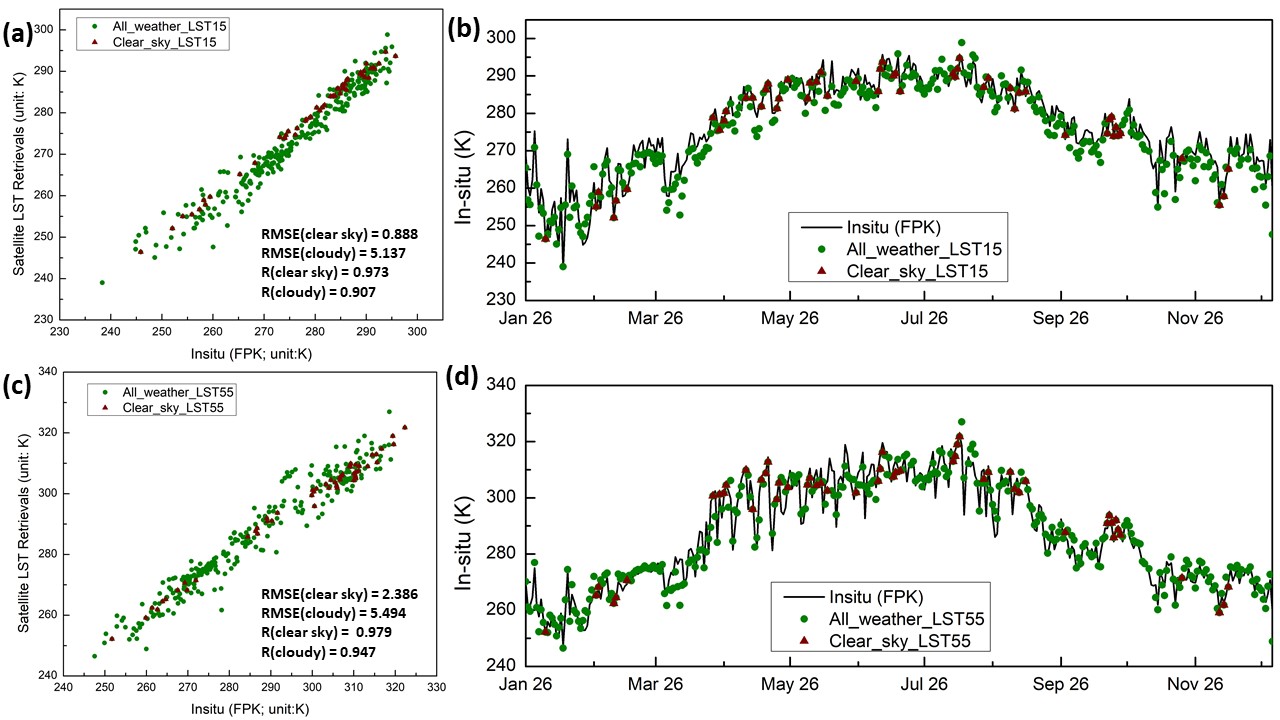


**Supplementary Figure 1.** Scatter plot (a,c) and time series (b,c) comparison of clear-sky LST (based on GOES16/17 only) and all-weather LST (based on combined GOES/AMSR2/CFSR), along with in-situ LST measurements over the SURFRAD-FPK station; (a-b) comparison at Time 1 (1.5 hour after sun rise) and (c-d) comparison at Time 2 (1.5 hour before noon); Unit: K.


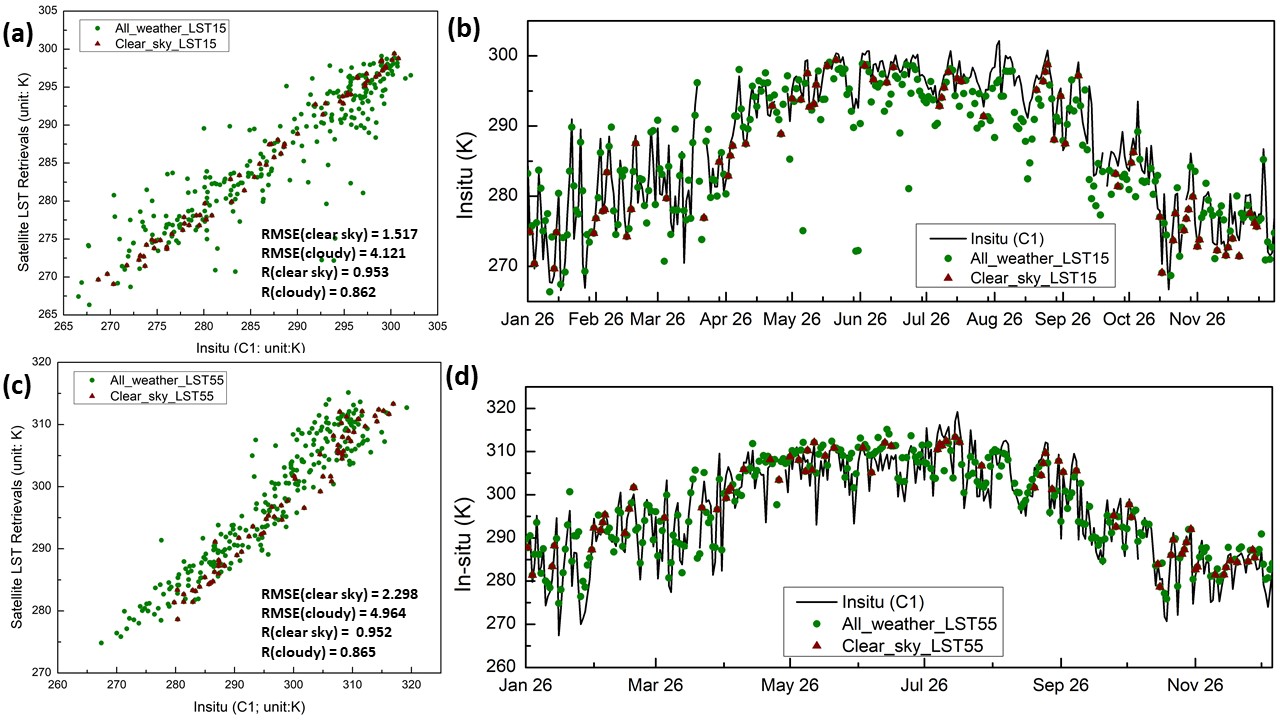


**Supplementary Figure 2.** Same as Figure 1, but for the ground station of C1


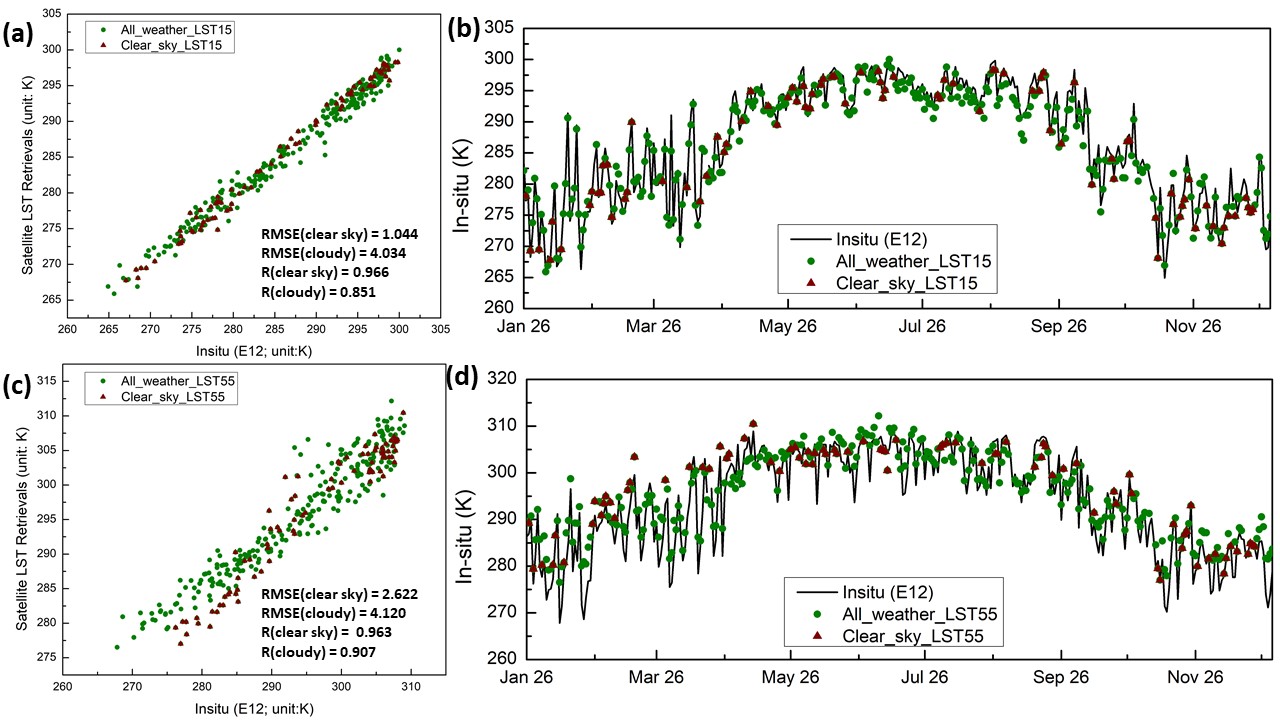


**Supplementary Figure 3.** Same as Figure 1, but for the ground station of E12


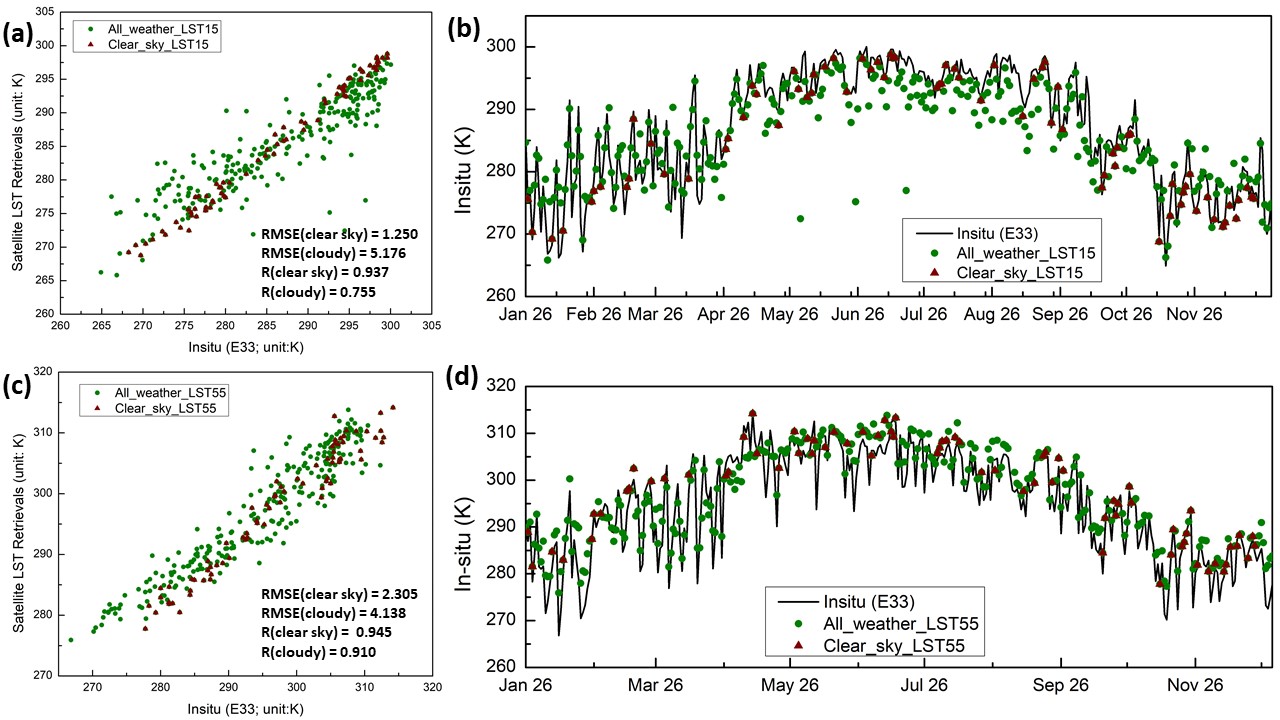


**Supplementary Figure 4.** Same as Figure 1, but for the ground station of E33


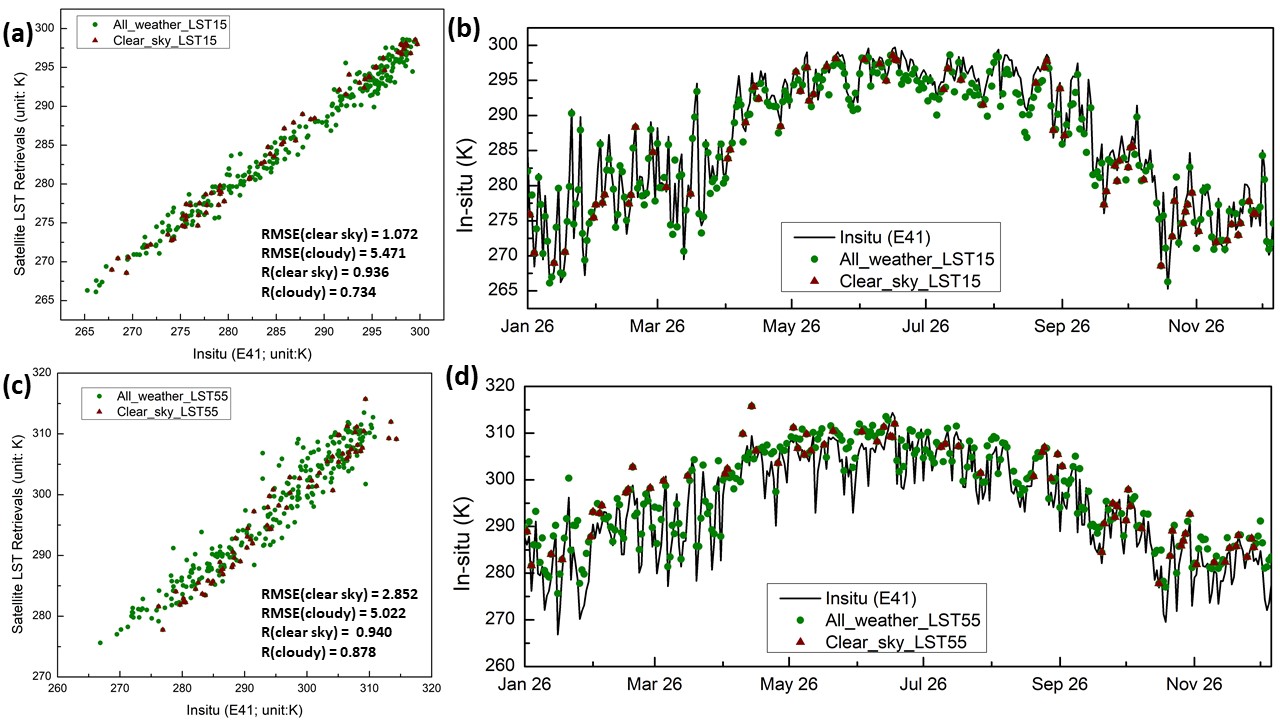


**Supplementary Figure 5.** Same as Figure 1, but for the ground station of E41


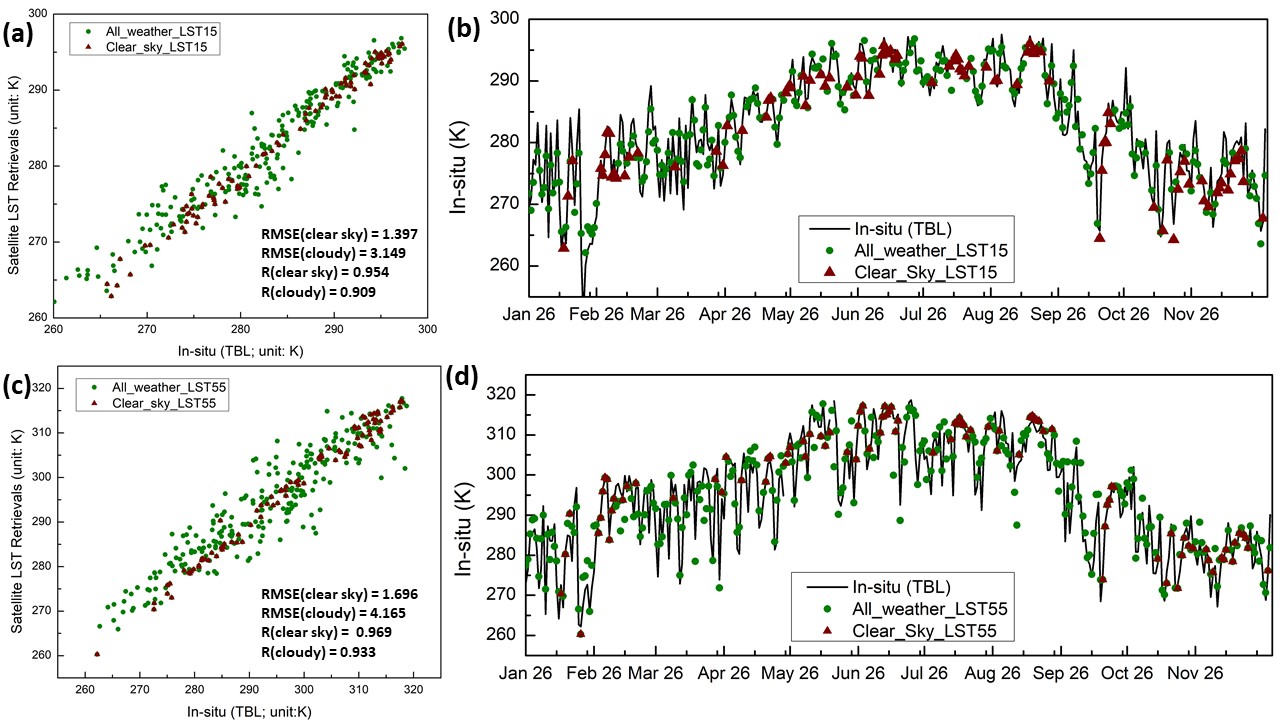


**Supplementary Figure 6.** Same as Figure 1, but for the ground station of TBL
